# The Medical Profession and the Notification of Births Act

**Published:** 1915-06-19

**Authors:** 


### The Hospital

The Workers' Newspaper of Administrative Medicine and Institutional Life, Administration, National Insurance and Health.

No. 1514, Vol. LVIII. SATURDAY, JUNE 19, 1915. PRICE ONE PENNY

### THE MEDICAL PROFESSION AND THE NOTIFICATION OF BIRTHS ACT.

Some of our readers will doubtless remember the controversies which arose when, in 1907, a Bill for the compulsory notification of births was introduced into Parliament. The proposal was—it is now the law of the land—that the birth of a child should be intimated in writing to the medical officer of health of the district not later than thirty-six hours after the occurrence of the event. The responsibility for the notification was placed primarily upon the father of the child should he be in residence at the time in the house where the birth took place, and, secondarily, upon any person in attendance upon the mother at the time of the birth or within six hours after its occurrence. Necessarily this latter enactment often involves a member of the medical profession, and the practitioner will not be excused for a failure to notify unless he can show that he had reasonable ground for believing that the notification had been effected by someone else. The translation of this into practice has meant, as was pointed out at the time it necessarily must mean, that the responsibility for notification falls upon the doctor. He has, in short, in this particular respect been made a kind of social policeman compelled to act on behalf of what are proclaimed to be the interests of the State. For this duty he does not receive any payment, though any neglect of it carries the risk of an appearance before a magistrate and a substantial money penalty. The one modification of these proposals effected by their opponents was the conversion of the Act from a compulsory into an optional one, its adoption or non-adoption in any individual district being left to the decision of the local authority. This is the position of affairs at the present moment; in some parts of the country the Act and all its consequences are in operation, while in others the Act has no application.

A suggestion has recently been advanced that the Notification of Births Act shall now be made of universal application over the length and breadth of the land, and that the option of the local authorities shall be cancelled. The motive of this proposal is the enhanced value of infant life as one of the assets of the State in consequence of the terrible losses which the war is inflicting on the manhood of the nation. The promise of babies enough and to spare which recently agitated the minds and meetings of social and moral reformers we are now told is not to be fulfilled. All that can be expected is the ordinary regular and respectable supply reinforced by such contributions as are usual and familiar even in times of peace. The war-babies are no more real than were the earlier and adventurous Russians. In such circumstances, naturally and properly, there is increased emphasis placed upon the importance of the newly arriving generation, and, other considerations apart, there are patriotic reasons why every effort should be made to secure favourable conditions for the early steps of its several members. In so far as the Notification of Births Act helps to secure this end the medical profession has no word to say against it. The care of infant life and its successful development have been matters of concern to the profession long ere Parliamentary reformers announced themselves upon the scene.

In view of the proposed extension of the existing Act in the direction above indicated it may be well briefly to re-state the medical objections to the original scheme. These objections remain unchanged, and may be reasonably advanced either when the question is re-discussed in Parliament or when a proposal is made in any locality to adopt the Act as it stands at the present moment. The centre of opposition is the necessity which is laid on the practitioner to reveal information which he has gained relative to his patient while acting in his professional capacity. It may be advisable or even necessary in the interests of the State that a birth shall be notified within thirty-six hours of its occurrence. To gain this end it may be quite appropriate that the father or some other person acting for him shall be compelled to convey the information to the officer appointed for the purpose. Upon these propositions we offer no dispute. What we do say is that the medical practitioner should not fall under this compulsion. For this contention there are good and substantial reasons. First, the practitioner is employed and paid not by the State, but by the individual patient. It is to the latter, therefore, and not to the former that his obligations are due. Still more important is the consideration that the confidence of the public in the silence of the medical profession is a large general interest. Whatever tends to weaken this means as an inevitable consequence the concealment in greater or less degree of the early, and therefore curable, stages of disease and the resort of sufferers to unqualified and unrestrained practitioners. To compel a medical man as he leaves his patient's house to announce to a public official the events of which he has just been a witness cannot fail to weaken confidence in his value as a respecter of the secrets entrusted to his keeping. It is therefore, we contend, a proceeding not merely obnoxious to the individual, but is, in addition, hostile to the public welfare. By all means let the State insist on whatever information it needs, but let it procure this information without an invasion of the confidential relations which in the general interest exist between the medical practitioner and his patient. This sound principle the Notification of Births Act ignores, and for this reason it merits the sustained opposition alike of the profession and of the public.

